# OsBSK1-2, an Orthologous of AtBSK1, Is Involved in Rice Immunity

**DOI:** 10.3389/fpls.2017.00908

**Published:** 2017-06-21

**Authors:** Jing Wang, Hui Shi, Lian Zhou, Chunfang Peng, Dingyou Liu, Xiaogang Zhou, Wenguan Wu, Junjie Yin, Hai Qin, Weiwei Ma, Min He, Weitao Li, Jichun Wang, Shigui Li, Xuewei Chen

**Affiliations:** ^1^State Key Laboratory of Hybrid Rice, Key Laboratory of Major Crop Diseases & Collaborative Innovation Center for Hybrid Rice in Yangtze River Basin, Sichuan Agricultural University at Wenjiang, ChengduChina; ^2^Rice Research Institute, Agricultural Academy of Sciences at Mianyang, MianyangChina

**Keywords:** OsBSK, PTI, rice blast, brassinosteroids, rice

## Abstract

The brassinosteroid-SIGNALING KINASE (BSK) belongs to the receptor-like cytoplasmic kinase XII subgroup. BSK1 regulates development and immunity in *Arabidopsis*. However, the function of rice (*Oryza sativa*) BSK1 is largely unknown. Here, we report that the expression level of *OsBSK1-2* is induced after a chitin or fagellin22 (flg22) treatment. Silencing *OsBSK1-2* in rice results in compromised responses to chitin- or flg22-triggered immunity and resistance to *Magnaporthe oryzae*, but does not alter the plant’s architecture nor reduce plant responses to brassinosteroid signaling. Our study reveals that OsBSK1-2 functions as a major regulator in rice plant immunity.

## Introduction

Receptor-like cytoplasmic kinases (RLCKs) are a subgroup of the receptor-like kinase family, but unlike the receptor-like kinases, which have an extracellular receptor domain, a transmembrane domain, and an intracellular kinase domain (Shiu and Bleecker, 2001), RLCKs just have a typical catalytic kinase, and they localize to the cytoplasm. Several RLCKs are predicted as regulators of plant development and immunity (Vij et al., 2008).

Receptor-like cytoplasmic kinases VII is an important gene subfamily in plant. In *Arabidopsis*, BOTRYTIS INDUCED KINASE 1 (BIK1) was first isolated as an early-induced kinase in response to infection by *Botrytis cinerea* (Veronese et al., 2006). BIK1 was then revealed to play an essential role in regulating immune responses mediated by pattern recognition receptors (PRRs), including flagellin-sensitive-2 (FLS2), EF-Tu receptor (EFR), and PEP-RECEPTOR1 (PEPR1) (Zhang et al., 2010; Eckardt, 2011; Liu et al., 2013). The orthologs of BIK1, PBS1, PBL1, PBL2 and PBL27, were also identified as regulators for pattern-triggered immunity (PTI) (Zhang et al., 2010; Lin et al., 2013; Liu et al., 2013; Yamaguchi et al., 2013a,b; Shinya et al., 2014). Other members of the RLCK VII subfamily have also been characterized as regulators in rice immunity. For examples, OsRLCK185, which is phosphorylated by OsCERK1 upon recognition of chitin and then dissociates from the OsCERK1 complex to activate MAPK cascades, is involved in an immune response (Yamaguchi et al., 2013a,b). OsRLCK57, OsRLCK107, OsRLCK118 and OsRLCK176, four homologs of BIK1, positively regulate immune responses by contributing to the expression of the immune receptor XA21 (Zhou et al., 2016).

Recently, brassinosteroid (BR)-signaling kinase1 (BSK1), a member of RLCK-XII, which belongs to another RLCK subfamily, was reported to play vital roles in plant immunity. BSK1, first isolated as a phosphorylation substrate of the BR receptor kinase BRI1, plays critical role in BR signaling (Tang et al., 2008). In *Arabidopsi*s, 12 BSK members were predicted (BSK1–12). Biochemical assays revealed that BRI1 targets many BSKs for phosphorylation, including BSK1, BSK3, BSK5, BSK6 and BSK11, suggesting that these BSKs might have redundant functions in response to BRs (Tang et al., 2008; Sreeramulu et al., 2013). Moreover, the *bsk1* mutant showed compromised resistance to various pathogens and responses to PTI. BSK1 associates with the PRR FLAGELLIN SENSING2 (FLS2) to activate downstream signal transduction in plant immunity (Shi et al., 2013). In rice, a member of the RLCK-XII subgroup, OsBSK3, plays a positive role in BR signaling, which can be phosphorylated by OsBRI1 and then associates with BRI1 SUPPRESSOR1 to transduce the BR signal (Zhang et al., 2016). However, the biological roles of their functions in rice immunity are poorly known.

In this study, we report that the enrichment of the transcripts levels of *OsBSK1-2* was induced by both flg22 and chitin. Silencing *OsBSK1-2* reduced the transcriptional expression of the pathogenesis-related genes, *Os04g10010* and *OsPR10b*, in response to the treatment of flg22 or chitin and compromised plant resistance to *Magnaporthe oryzae*. However, plants with reduced *OsBSK1-2* expression levels did not alter plant architecture or BR signaling. Thus, our study reveals that OsBSK1-2 functions as a positive regulator in rice immunity.

## Materials and Methods

### Plant Growth

All of the transgenic plants were created in the ‘Kitaake’ background. All of the plants were grown in a controlled rice field in Chengdu, Sichuan, China.

### Construction

All of the primers used here are listed in Supplementary Table S1. To generate RNA interference (RNAi) construction, the unique cDNA sequence of *OsBSK1-2* was amplified and cloned into the pCRR8^TM^/GW/TOPO^®^ (Invitrogen, Carlsbad, CA, United States) vector to create the pCR8-*OsBSK1-2Ri* constructs. Then, the RNAi fragments were inserted into the pANDA vector (Miki and Shimamoto, 2004) using LR recombination to create the RNAi construct pANDA-*OsBSK1-2Ri* (*OsBSK1-2Ri*).

### Generation of Transgenic Rice

*OsBSK1-2Ri* was introduced into ‘Kitaake’ plants through *Agrobacterium*-mediated transformation, and hygromycin was used to select the regenerated plants (Chern et al., 2005).

### RNA Extraction and Quantitative Real Time RT-PCR Analyses

Total RNA was prepared from samples collected using Trizol (Invitrogen) according to the manufacturer’s instructions. The RNA samples were then subjected to cDNA synthesis system with RT reagent Kit with gDNA Eraser (Takara, Dalian, China). A Quantitative RT-PCR Kit was used to establish the qRT-PCR reactions (Qiagen, Valencia, CA, United States). The gene expression levels were normalized to the transcript level of the reference gene *ubiquitin5* (*LOC_06g46770*) gene. All of the primer pairs used for qRT-PCR, which are listed in Supplementary Table S2, have been tested for efficiency and specificity following the guidelines for successful real time^1^. qRT-PCR analysis were performed follow the MIQe guidelines (Bustin et al., 2009).

### Defense Gene Expression Analysis

The 2–3 cm long leaf strips from fully developed leaves of 4-week-old seedlings were incubated for 12 h in ddH_2_O. They were then treated with 1 μM flg22 peptide (Pacific Immunology, San Diego, CA, United States) or 20 μg/ml chitin from shrimp shells (Sigma, St. Louis, MO, United States) according to a method described previously (Felix et al., 1999). The samples were collected after 12 h for further investigation.

### Brassinolide (BL) Treatment

To analyze the effects of BL on the lamina joint angles of the transgenic plants, 1 μl 2,4-epiBL (100 ng/μl) was applied to the leaf angles and the lamina joint angles were measured following a method described previously (Chen et al., 2013).

### Phylogenetic and Molecular Evolutionary Analyses

To identify ortholog(s) of AtBSK1 in the rice genome, the amino acid sequence of AtBSK1 was used as the seed sequence to perform a BLAST algorithm-related search of the rice protein database^2^. The proteins with hit scores more than 1,000 and E-value less than 1.6e^-100^, were chosen for further study. Twelve AtBSK1 and five OsBSKs were first aligned using ClusterX software. Then, a phylogenetic tree was constructed using MEGA5.10 software. The evolutionary history was inferred using the Maximum Likelihood method based on the Poisson correction model. The tree with the highest log likelihood (-6702.6606) is shown. Initial tree(s) for the heuristic search were obtained automatically by applying Neighbor-Joining and BioNJ algorithms to a matrix of pairwise distances estimated using a JTT model, and then selecting the topology with superior log likelihood value. The tree is drawn to scale, with branch lengths measured using the number of substitutions per site (Tamura et al., 2011).

### Rice Blast Incubation

*Magnaporthe oryzae* isolates, ZHONG1 and ZB25, were used for the inoculations. Leaf strips from 8-week-old plants were lightly wounded with a mouse ear punch, and 5 μl of spore suspension (5 × 10^5^ spores/ml) was added to the wound. The lesion size was measured after incubating for 8–14 days at 28°C. The DNA was then extracted to calculate the expression level of the *Pot2* gene of *M. oryzae* relative to the genomic *Ubiquitin* in rice (Park et al., 2012).

## Results

### Characterization of OsBSK1-1 and OsBSK1-2

**FIGURE 1:**
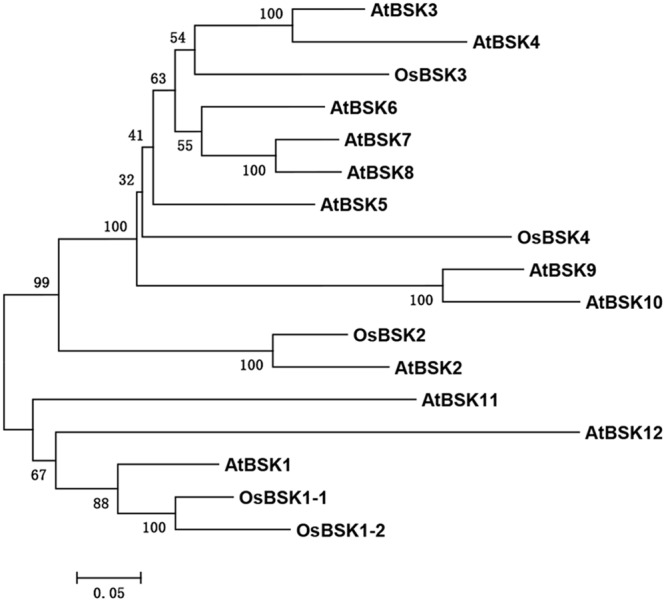
Phylogenetic analysis on five OsBSKs and 12 *Arabidopsis* BSKs. Five OsBSKs and 12 AtBSKs were subjected to the ClustalX analysis with default parameters. Phylogenetic was built using MEGA version5.10. Two orthologous of AtBSK1 were identified as OsBSK1-1 and OsBSK1-2.

Many BR signaling partners have been characterized in rice, and they have functions that are similar to those of their orthologs in *Arabidopsis* (Tong and Chu, 2012). AtBSK1 was reported to regulate *Arabidopsis* immunity. We therefore presumed that the rice ortholog(s) of AtBSK1 should function in rice immunity. To test this hypothesis, we identified five OsBSKs, OsBSK1-1 (LOC_Os03g04050), OsBSK1-2 (LOC_Os10g39670), OsBSK2 (LOC_Os10g42110), OsBSK3 (LOC_Os04g58750), and OsBSK4 (LOC_Os03g61010) from rice genome by blast research using the amino acid sequence of AtBSK1 as the seed sequence. The result showed that two proteins, OsBSK1-1 (LOC_Os03g04050) and OsBSK1-2 (LOC_Os10g39670) which share more than 79% similarity with each other in amino acid sequence were sub-grouped with AtBSK1 in phylogenetic and molecular evolutionary analyses (**Figure 1**).

**FIGURE 2:**
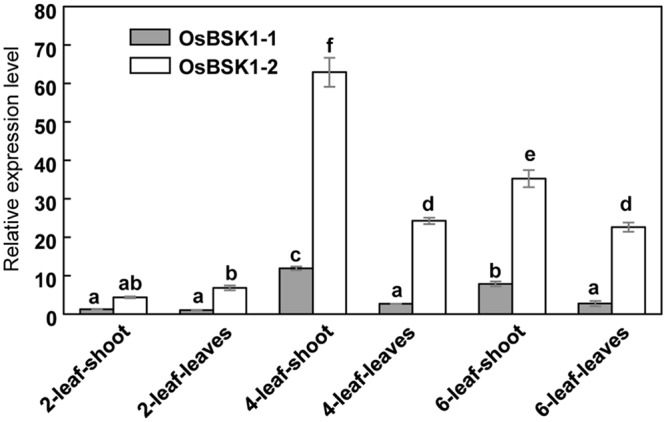
Determination of the expression patterns of *OsBSK1-1* and *OsBSK1-2*. qRT-PCR analysis was performed on cDNA synthesized from RNA samples. The RNA samples were extracted from tissues of rice cultivar Kitaake at the development stages as indicated. The expressions of the *OsBSK1-1* and *OsBSK1-2* were normalized to level of the *ubiquitin* reference gene. Error bars indicate SD obtained from three technical replicates. The letters indicate significant differences as determined by a one-way ANOVA followed by *post hoc* Tukey HSD analysis.

To determine the expression patterns of *OsBSK1-1* and *OsBSK1-2*, we measured their transcription levels in different developmental stages using quantitative reverse transcription-PCR (qRT-PCR). Both *OsBSK1-1* and *OsBSK1-2* were predominantly expressed in the four-leaf stage of development, and the transcripts of *OsBSK1-2* were more abundant than those of *OsBSK1-1* (**Figure 2**).

### Silencing *OsBSK1-2* Inhibits Flagellin- and Chitin-Triggered Immune Responses in Rice

**FIGURE 3:**
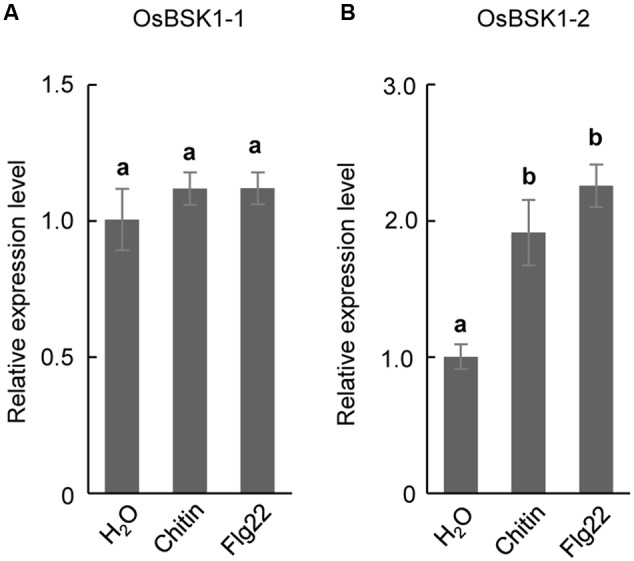
The expression of *OsBSK1-2* is induced by PAMPs. The expression analyses on *OsBSK1-2* **(A)** and *OsBSK1-1* **(B)**, respectively, in rice leaves underflg22- and chitin- treatments. The Results from two independent biological experiments were combined with statistical analysis. The letters indicate significant differences determined by a one-way ANOVA followed by *post hoc* Tukey HSD analysis.

BSK1 associates with FLS2 to regulate plant PTI in *Arabidopsis* (Shi et al., 2013). To test whether OsBSK1-1 and OsBSK1-2 regulate PTI in rice, we collected leaf strips that were 2–3 cm in length from fully developed leaves of 4-week-old Kitaake seedlings and treated them with flg22 peptide or chitin. Then, we detected the transcription levels of *OsBSK1-1* and *OsBSK1-2*, and found that *OsBSK1-2* was induced after the flg22 and chitin treatments, while *OsBSK1-1* was not (**Figure 3**). We then chose *OsBSK1-2* for further studies.

To analyze whether *OsBSK1-2* functions in rice immunity, we generated *OsBSK1-2* RNAi transgenic rice lines. Four independent transgenic lines (*OsBSK1-2*Ri) with reduced expression levels of *OsBSK1-2* were obtained (**Supplementary Figure S1**).

**FIGURE 4:**
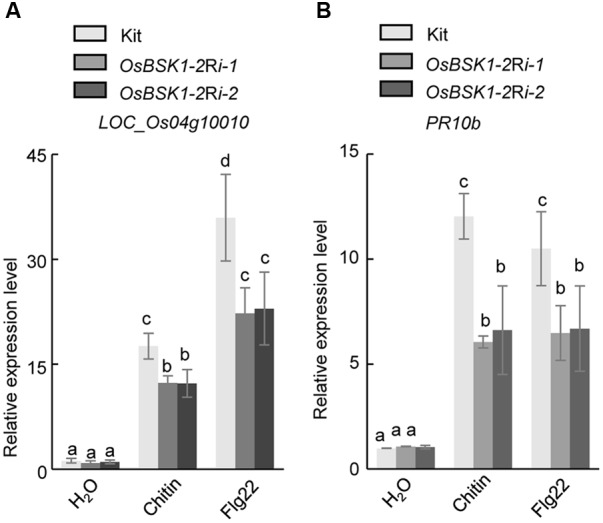
Effects of silencing *OsBSK1-2* on flg22- and chitin-triggered immune responses. Leaf strips of 4-week-old plants were treated with 1 μM flg22 peptide (Pacific immunology) or 20 ug/ml chitin (Sigma) for 12 h. The mock treatment was ddH_2_O. The expression levels of two defense marker genes -*Os04g10010* **(A)** and -*OsPR10b* **(B)** were measured by qRT-PCR. Gene expression levels for the target gene were normalized to the *ubiquitin* gene. Data shown were normalized to the Kitaake mock-treated (12 h) samples (100%). The Results from two independent biological experiments were combined with statistical analysis. The letters indicate significant differences (one-way ANOVA followed by *post hoc* Tukey HSD analysis).

The genes *Os04g10010* and *OsPR10b* function as markers involved in the downstream responses associated with PTI (Park et al., 2012; Chen et al., 2014). We thus examined the transcription levels of these two genes in *OsBSK1-2Ri* transgenic plants after the flg22 or chitin treatments. In Kitaake plants, the expressions of *Os04g10010* and *OsPR10b* were induced by 17.0 ± 0.9- and 10.3 ± 0.6-fold, respectively, after the chitin treatment (**Figure 4**). However, the expression levels of *Os04g10010* and *OsPR10b* were induced only about 12.7 ± 0.7- and 5.1 ± 0.8-fold in *OsBSK1-2*Ri-1 and 10.6 ± 0.7- and 4.2 ± 0.5-fold in *OsBSK1-2*Ri-2 plants, respectively. Similarly, the expression levels of *Os04g10010* and *OsPR10b* were induced only about 25.6 ± 0.1- and 8.2 ± 0.1-fold in *OsBSK1-2*Ri-1 and 26.1 ± 2.2- and 8.6 ± 0.8-fold in *OsBSK1-2*Ri-2 plants, respectively, whereas induced about 41.0 ± 2.1- and 12.4 ± 0.2-fold in the wild type Kitaake after the flg22 treatment (**Figure 4**). Taken together, these results suggest that the silencing of OsBSK1-2 compromises the base immune response in rice. Thus, OsBSK1-2 might be involved in the regulation of rice PTI.

### Silencing of *OsBSK1-2* Compromises Plant Resistance to *M. oryzae*

**FIGURE 5:**
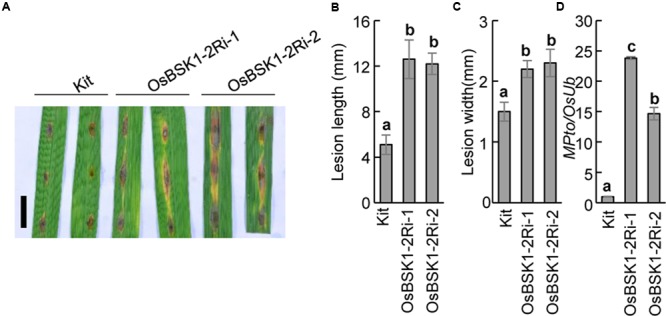
Silencing of *OsBSK1-2* compromises plant resistance to *Magnaporthe oryzae*. The leaf strips from 8-week-old plants were inoculated the suspension of *M. oryzae* isolate ZB25 after 12 h post lightly wounding. **(A)** Photographs of rice leaves 10 days after *M. oryzae* inoculated. Bars = 10 mm. The length **(B)** and width **(C)** of lesion size were measured 10 days after *M. oryzae* inoculated (*n* = 10). **(D)** The relative fungal growth was determined on the inoculated leaves. Gene expression levels for the target gene were normalized to the *ubiquitin* gene (*n* = 3). The letters indicate significant differences as determined by a one-way ANOVA followed by *post hoc* Tukey HSD analysis. Three independent biological repeats were performed and similar results were obtained.

To further investigate the genetic role of *OsBSK1-2* in rice immunity, we inoculated the *OsBSK1-2*Ri transgenic and wild type plants with the *M. oryzae* isolate ZB25. Ten days post-inoculation, the lesion size of *OsBSK1-2*Ri-1 (12.6 ± 1.7 cM for lesion length and 2.2 ± 0.1 cM for lesion width) and *OsBSK1-2*Ri-2 (12.2 ± 1.0 cM and 2.3 ± 0.2 cM) plants displayed larger lesions than the Kitaake plants (5.1 ± 0.9 cM and 1.5 ± 0.2 cM) (**Figures 5A–C**). To determine the fungal biomass in the inoculated leaves precisely, we isolated the total DNA from the infected leaves and quantified it using a DNA-based quantitative PCR method (Park et al., 2012). Relative to the reference rice *Ubiquitin* gene, the DNA accumulation level of fungal gene *Pto2* in *OsBSK1-2*Ri-1/-2 plants was higher than in Kitaake plants (**Figure 5D**). This result revealed that the fungal biomass was more accumulated in the infected *OsBSK1-2*Ri-1/-2 than in the wild type Kittake leaves. We then inoculated *OsBSK1-2*Ri and wild type plants with another compatible *M. oryzae* isolate, ZHONG1, and obtained similar results (**Supplementary Figure S2**).

To further confirm the results, we inoculated segregants derived from the *OsBSK1-2*Ri transgenic plants with the *M. oryzae* isolate ZB25 and determined the resistance. The segregants carrying the silencing constructs showed larger lesions than wild type Kittake, while segregants lacking the silencing constructs had a similar lesion size as the wild type (**Supplementary Figure S3**). Therefore, we concluded that silencing *OsBSK1-2* resulted in compromised rice resistance to *M. oryzae.*

### Silencing of *OsBSK1-2* Does Not Alter Plant Architecture or Sensitivity to BR

**FIGURE 6:**
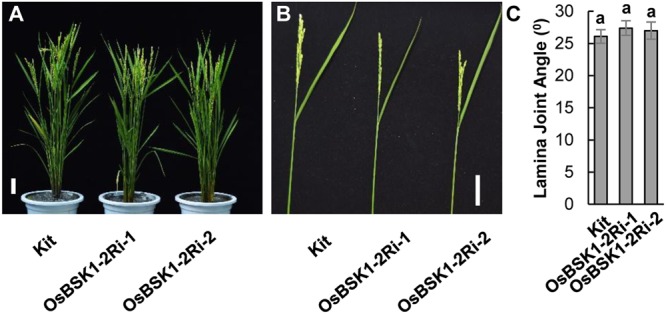
Plant architecture of Kitaake plants with reduced expressions of *OsBSK1-2*. **(A)** The gross morphological phenotypes of Kitaake and transgenic plants silenced for *OsBSK1-2*. Bars = 10 cm. **(B)** Photograph of a representative leaf of Kitaake and *OsBSK1-2*Ri plants. Bars = 5 cm. **(C)** Statistical analyses on the lamina joint angles of Kitaake and the plants silenced for *OsBSK1-2*. The averages and SDs were calculated from 10 plants of the representative transgenic *OsBSKRi* lines as indicated (*n* = 10). The letters indicate significant differences using a one-way ANOVA followed by *post hoc* Tukey HSD analysis.

To explore the biological role of OsBSK1-2 in plant development, we analyzed the plant architecture of *OsBSK1-2Ri* plants, including plant height or lamina joint. There were no obvious differences in plant height or lamina joint between *OsBSK1-2Ri* and Kittake plants (**Figure 6** and **Supplementary Figures S4A,B**).

**FIGURE 7:**
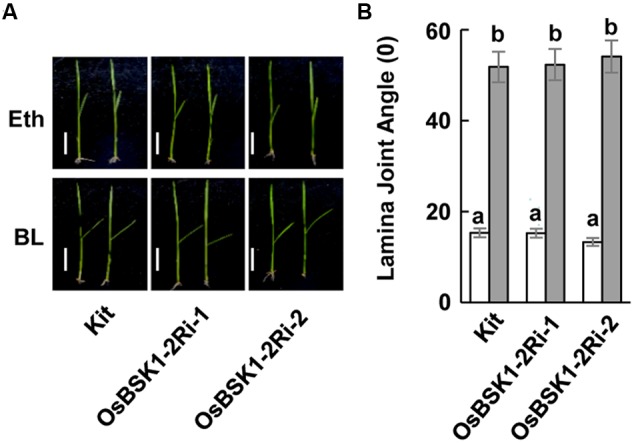
Effects of silencing the *OsBSK1-2* on plants in response to BL hormone. **(A)** Photographs of representative seedlings showing the leaf angles for the Kitaake (Kit) plants and *OsBSK1-2Ri* after the mock-treatments (Upper panel) or BL treatment (Lower panel). The BL treatment contained 2,4-epiBL (100 ng), whereas Eth alone was used for the mock treatment. **(B)** Statistical analysis of the leaf angles of Kitaake and *OsBSK1-2Ri* after BL and mock-treatments, respectively. Each bar represents the average and SD of 10 leaves. The letters indicate significant differences as determined by a one-way ANOVA followed by *post hoc* Tukey HSD analysis. Two independent repeats were performed with similar results produced.

To determine whether *OsBSK1-2* regulated plant responses to the hormone BR, we treated the lamina joint of the wild type and *OsBSK1-2Ri* plants with BR. The lamina joint of *OsBSK1-2Ri* plants was seriously enlarged after the BL treatment, which was similar to the wild type (**Figure 7** and **Supplementary Figure S4C**). These results suggest that silencing *OsBSK1-2* does not alter plant architecture or responses to BRs.

## Discussion

In *Arabidopsis*, a total of 12 BSKs have been isolated, and play various function in plant development and immunity (Tang et al., 2008; Sreeramulu et al., 2013). Although five OsBSKs identified in rice share a conserved kinase domain and a C-terminal tetratricopeptide repeat domain, their genetic functions in plant immunity and development might be different.

*OsBSK1-1* and *OsBSK1-2* are two homologs that also share similar expression patterns in rice developmental stages. However, the transcripts of *OsBSK1-2* are more abundant than those of *OsBSK1-1* in rice (**Figure 2**), suggesting that OsBSK1-2 might play more important roles in rice. In agreement, the expression level of *OsBSK1-2*, but not *OsBSK1-1*, is obviously induced after flg22 and chitin treatments (**Figure 4**). Therefore, OsBSK1-1 and OsBSK1-2 might play different roles in plant immunity. OsBSK1-2 is an ortholog of AtBSK1, which also regulate FLS2-mediated immunity as AtBSK1 (Shi et al., 2013). Although, no direct interaction between OsBSK1-2 and OsFLS2 was determined, OsBSK1-2 might still play a similar molecular mechanism as does AtBSK1 in plant immunity because silencing of *OsBSK1-2* also compromised the base immune response in rice (**Figure 4**).

Brassinosteroid-SIGNALING KINASE are important regulators of BR signaling (Tang et al., 2008; Sreeramulu et al., 2013). However, we did not observe the BR-related phenotypes in rice plants in silence of *OsBSK1-2.* BSK1 is phosphorylated by BRI1 upon BR perception and disassociates from BRI1 to activate BRI1 SUPPRESSOR1 in *Arabidopsis* (Tang et al., 2008; Kim et al., 2009). BRI1 interacts with and phosphorylates the BSKs, BSK1, BSK3, BSK5, BSK6, BSK8 and BSK11, to activate downstream signaling (Sreeramulu et al., 2013). Nevertheless, knocking out any individual *AtBSK* in *Arabidopsis* does not affect plant architecture because of their redundant functions (Sreeramulu et al., 2013). LOC_Os04g58750 (OsBSK3) interacts with OsBRI1 *in vivo*, and the over-expression of OsBSK3 increases the hypersensitivity of rice plants to the hormone BR (Zhang et al., 2016). Although the reduced expression of *OsBSK1-2* does not alter the BR-triggered response in rice plants (**Figure 4**), it does not rule out the possibility of its involvement in the regulation of BR-mediated signaling because of the possible redundancies of the other four OsBSKs. Thus, it needs to generate double or multiple knock-out mutants for the OsBSKs in rice to determine whether OsBSK1-2 regulates BR-mediated signaling and plant development for the future studies.

## Author Contributions

JW and XC conceived this study. HS, LZ, CP, Dingyou Liu, XZ, WW, JY, HQ, WM, JCW performed the experiments. JW, MH, WL, Sigui Li, and XC analyzed data. JW and XC wrote the manuscript. All authors approved the manuscript.

## Conflict of Interest Statement

The authors declare that the research was conducted in the absence of any commercial or financial relationships that could be construed as a potential conflict of interest.

## Footnotes

1 https://www.qiagen.com

2 http://rice.plantbiology.msu.edu/

## Supplementary Material

FIGURE S1 Characterization of the transgenic plants silenced for *OsBSK1-2*. The relative expression levels of *OsBSK1-2* **(A)** and *OsBSK1-1* **(B)**, were determined in the transgenic lines and the wild type Kitaake plants by qRT-PCR. All data were normalized to the reference *ubiqutin* gene. The average and SD for the relative expression of each line is shown. Data were obtained from three technical replicates. The letters indicate significant differences as determined by a one-way ANOVA followed by *post hoc* Tukey HSD analysis. Three independent biological experiments were performed and similar results were obtained.

FIGURE S2 Silencing of *OsBSK1-2* compromises plant resistance to *Magnaporthe oryzae*. The leaf strips from 8-week-old plants were inoculated with the suspension of *M. oryzae* isolate ZHONG1. **(A)** Photographs of rice leaves 10 days after *M. oryzae* inoculation. Bars = 10 mm. The length **(B)** and width **(C)** of lesion size are measured 10 days after *M. oryzae* inoculated (*n* = 10). The letters indicate significant differences, one-way ANOVA followed by *post hoc* Tukey HSD analysis.

FIGURE S3 Co-segregation analysis on reduced resistance with the *OsBSK1-2Ri* transgene in plants. Segregants from the T_1_ progeny of transgenic *OsBSK1-2Ri-1* line was analyzed for disease resistance post the inoculation with *M. oryzae* isolates, ZB25 **(A)** and ZHONG1 **(B)**, respectively.

FIGURE S4 Silencing of *OsBSK1-2* does not effect plant response to BRs obviously. **(A)** The gross morphological phenotypes of Kitaake and transgenic plants silenced for *OsBSK1-2*. Bars = 10 cm. **(B)** Photograph of a representative leaf of Kitaake (Kit) and *OsBSK1-2*Ri plants. Bars = 5 cm. **(C)** Photographs of representative seedlings showing the leaf angles for the Kitaakeand *OsBSK1-2Ri* plants after the mock-treatments (Upper panel) or BL treatment (Lower panel). Eth solution containing 2,4-epiBL (100 ng) was used for the BL treatment, whereas Eth alone was used for the mock treatment.

## References

[B1] Bustin S. A. Benes V. Garson J. A. Hellemans J. Huggett J. Kubista M. (2009). The MIQE guidelines: minimum information for publication of quantitative real-time PCR experiments. *Clin. Chem.* 55 611–622. 10.1373/clinchem.2008.112797 19246619

[B2] Chen L. Xiong G. Cui X. Yan M. Xu T. Qian Q. (2013). OsGRAS19 may be a novel component involved in the brassinosteroid signaling pathway in rice. *Mol. Plant* 6 988–991. 10.1093/mp/sst027 23389891

[B3] Chen X. Zuo S. Schwessinger B. Chern M. Canlas P. E. Ruan D. (2014). An XA21-associated kinase (OsSERK2) regulates immunity mediated by the XA21 and XA3 immune receptors. *Mol. Plant* 7 874–892. 10.1093/mp/ssu003 24482436 PMC4064043

[B4] Chern M. S. Canlas P. E. Fitzgerald H. Ronald P. C. (2005). Rice NRR, a negative regulator of disease resistance in rice that interacts with Arabidopsis NPR1 and Rice NH1. *Plant J.* 43 623–635. 10.1111/j.1365-313X.2005.02485.x 16115061

[B5] Eckardt N. A. (2011). BIK1 function in plant growth and defense signaling. *Plant Cell* 23:2806 10.1105/tpc.111.230811 PMC3180790 21862709

[B6] Felix G. Duran J. D. Volko S. Boller T. (1999). Plants have a sensitive perception system for the most conserved domain of bacterial flagellin. *Plant J.* 18 265–276. 10.1046/j.1365-313X.1999.00265.x 10377992

[B7] Kim T. W. Guan S. Sun Y. Deng Z. Tang W. Shang J. X. (2009). Brassinosteroid signal transduction from cell-surface receptor kinases to nuclear transcription factors. *Nat. Cell Biol.* 11 1254–1260. 10.1038/ncb1970 19734888 PMC2910619

[B8] Lin W. Lu D. Gao X. Jiang S. Ma X. Wang Z. (2013). Inverse modulation of plant immune and brassinosteroid signaling pathways by the receptor-like cytoplasmic kinase BIK1. *Proc. Natl. Acad. Sci. U.S.A.* 110 12114–12119. 10.1073/pnas.1302154110 23818580 PMC3718091

[B9] Liu Z. Wu Y. Yang F. Zhang Y. Chen S. Xie Q. (2013). BIK1 interacts with PEPRs to mediate ethylene-induced immunity. *Proc. Natl. Acad. Sci. U.S.A.* 110 6205–6210. 10.1073/pnas.1215543110 23431184 PMC3625333

[B10] Miki D. Shimamoto K. (2004). Simple RNAi vectors for stable and transient suppression of gene function in rice. *Plant Cell Physiol.* 45 490–495. 10.1093/pcp/pch048 15111724

[B11] Park C. H. Chen S. Shirsekar G. Zhou B. Khang C. H. Songkumarn P. (2012). The *Magnaporthe oryzae* effector AvrPiz-t targets the RING E3 ubiquitin ligase APIP6 to suppress pathogen-associated molecular pattern-triggered immunity in rice. *Plant Cell* 24 4748–4762. 10.1105/tpc.112.105429 23204406 PMC3531864

[B12] Shi H. Shen Q. Qi Y. Yan H. Nie H. Chen Y. (2013). BR-SIGNALING KINASE1 physically associates with FLAGELLIN SENSING2 and regulates plant innate immunity in *Arabidopsis*. *Plant Cell* 25 1143–1157. 10.1105/tpc.112.107904 23532072 PMC3634682

[B13] Shinya T. Yamaguchi K. Desaki Y. Yamada K. Narisawa T. Kobayashi Y. (2014). Selective regulation of the chitin-induced defense response by the Arabidopsis receptor-like cytoplasmic kinase PBL27. *Plant J.* 79 56–66. 10.1111/tpj.12535 24750441

[B14] Shiu S. H. Bleecker A. B. (2001). Receptor-like kinases from *Arabidopsis* form a monophyletic gene family related to animal receptor kinases. *Proc. Natl. Acad. Sci. U.S.A.* 98 10763–10768. 10.1073/pnas.181141598 11526204 PMC58549

[B15] Sreeramulu S. Mostizky Y. Sunitha S. Shani E. Nahum H. Salomon D. (2013). BSKs are partially redundant positive regulators of brassinosteroid signaling in Arabidopsis. *Plant J.* 74 905–919. 10.1111/tpj.12175 23496207

[B16] Tamura K. Peterson D. Peterson N. Stecher G. Nei M. Kumar S. (2011). MEGA5: molecular evolutionary genetics analysis using maximum likelihood, evolutionary distance, and maximum parsimony methods. *Mol. Biol. Evol.* 28 2731–2739. 10.1093/molbev/msr121 21546353 PMC3203626

[B17] Tang W. Kim T. W. Oses-Prieto J. A. Sun Y. Deng Z. Zhu S. (2008). BSKs mediate signal transduction from the receptor kinase BRI1 in Arabidopsis. *Science* 321 557–560. 10.1126/science.1156973 18653891 PMC2730546

[B18] Tong H. Chu C. (2012). Brassinosteroid signaling and application in rice. *J. Genet. Genomics* 39 3–9. 10.1016/j.jgg.2011.12.001 22293112

[B19] Veronese P. Nakagami H. Bluhm B. Abuqamar S. Chen X. Salmeron J. (2006). The membrane-anchored *BOTRYTIS-INDUCED KINASE1* plays distinct roles in *Arabidopsis* resistance to necrotrophic and biotrophic pathogens. *Plant Cell* 18 257–273. 10.1105/tpc.105.035576 16339855 PMC1323497

[B20] Vij S. Giri J. Dansana P. K. Kapoor S. Tyagi A. K. (2008). The receptor-like cytoplasmic kinase (OsRLCK) gene family in rice: organization, phylogenetic relationship, and expression during development and stress. *Mol. Plant* 1 732–750. 10.1093/mp/ssn047 19825577

[B21] Yamaguchi K. Yamada K. Ishikawa K. Yoshimura S. Hayashi N. Uchihashi K. (2013a). A receptor-like cytoplasmic kinase targeted by a plant pathogen effector is directly phosphorylated by the chitin receptor and mediates rice immunity. *Cell Host Microbe* 13 347–357. 10.1016/j.chom.2013.02.007 23498959

[B22] Yamaguchi K. Yamada K. Kawasaki T. (2013b). Receptor-like cytoplasmic kinases are pivotal components in pattern recognition receptor-mediated signaling in plant immunity. *Plant Signal. Behav.* 8:e25662 10.4161/psb.25662 PMC4091068 23857358

[B23] Zhang B. Wang X. Zhao Z. Wang R. Huang X. Zhu Y. (2016). OsBRI1 activates BR signaling by preventing binding between the TPR and kinase domains of OsBSK3 via phosphorylation. *Plant Physiol.* 170 1149–1161. 10.1104/pp.15.01668 26697897 PMC4734578

[B24] Zhang J. Li W. Xiang T. Liu Z. Laluk K. Ding X. (2010). Receptor-like cytoplasmic kinases integrate signaling from multiple plant immune receptors and are targeted by a *Pseudomonas syringae* effector. *Cell Host Microbe* 7 290–301. 10.1016/j.chom.2010.03.007 20413097

[B25] Zhou X. Wang J. Peng C. Zhu X. Yin J. Li W. (2016). Four receptor-like cytoplasmic kinases regulate development and immunity in rice. *Plant Cell Environ.* 39 1381–1392. 10.1111/pce.12696 26679011

