# Supplementary figures and images for: OsBSK1-2, an Orthologous of AtBSK1, Is Involved in Rice Immunity
Source: Front Plant Sci. 2017 Jun 21;8:908. doi: 10.3389/fpls.2017.00908 (PMC5478731; doi:10.3389/fpls.2017.00908)

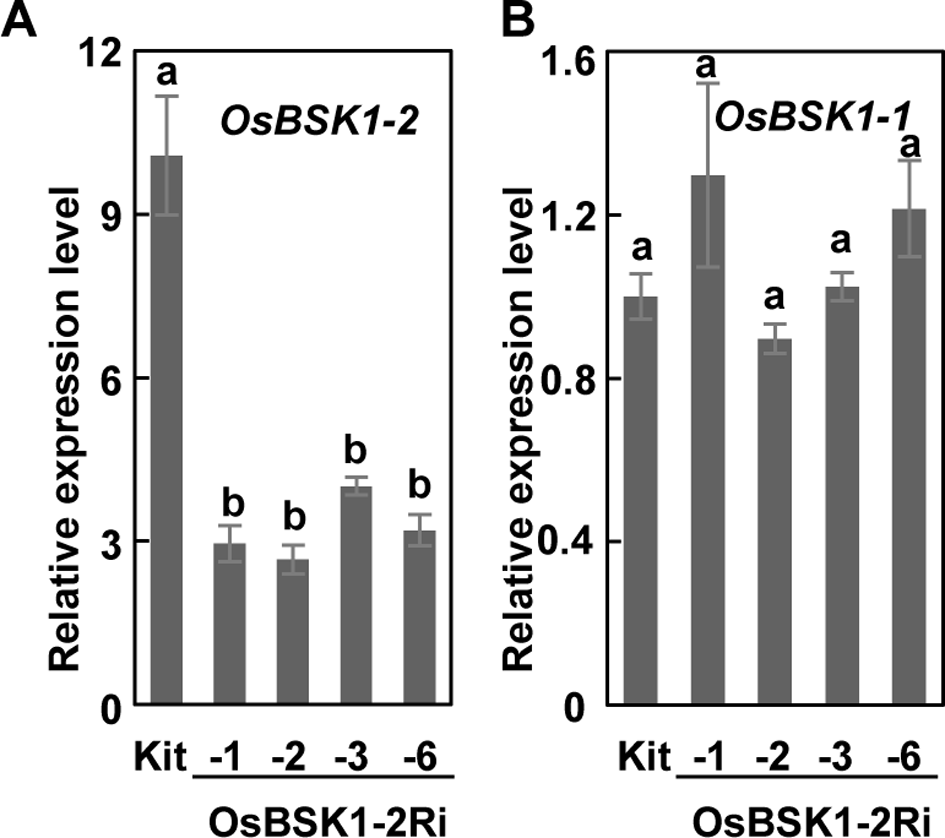

Supplement: FIGURE S1 — Characterization of the transgenic plants silenced for OsBSK1-2. The relative expression levels of OsBSK1-2 (A) and OsBSK1-1 (B), were determined in the transgenic lines and the wild type Kitaake plants by qRT-PCR. All data were normalized to the reference ubiqutin gene. The average and SD for the relative expression of each line is shown. Data were obtained from three technical replicates. The letters indicate significant differences as determined by a one-way ANOVA followed by post hoc Tukey HSD analysis. Three independent biological experiments were performed and similar results were obtained. [file Image_1.TIF]

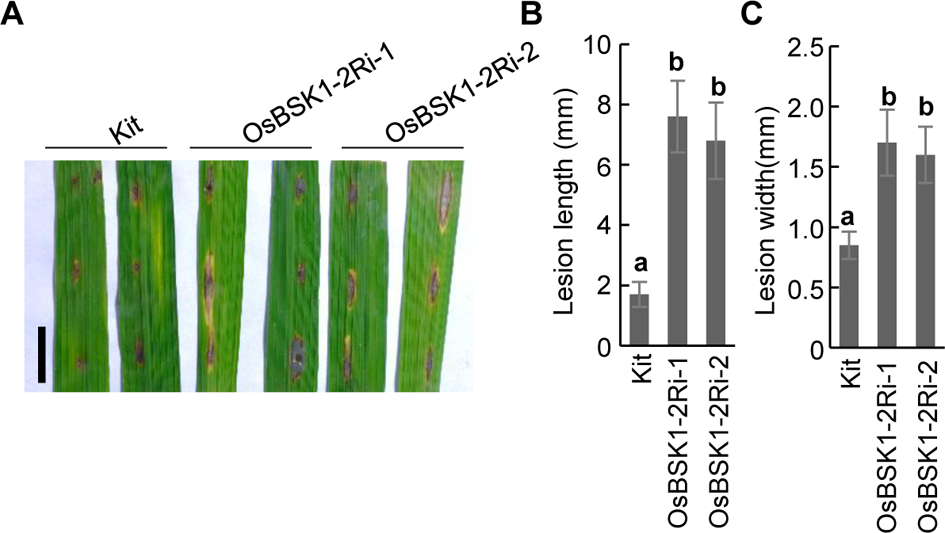

Supplement: FIGURE S2 — Silencing of OsBSK1-2 compromises plant resistance to Magnaporthe oryzae. The leaf strips from 8-week-old plants were inoculated with the suspension of M. oryzae isolate ZHONG1. (A) Photographs of rice leaves 10 days after M. oryzae inoculation. Bars = 10 mm. The length (B) and width (C) of lesion size are measured 10 days after M. oryzae inoculated (n = 10). The letters indicate significant differences, one-way ANOVA followed by post hoc Tukey HSD analysis. [file Image_2.tif]

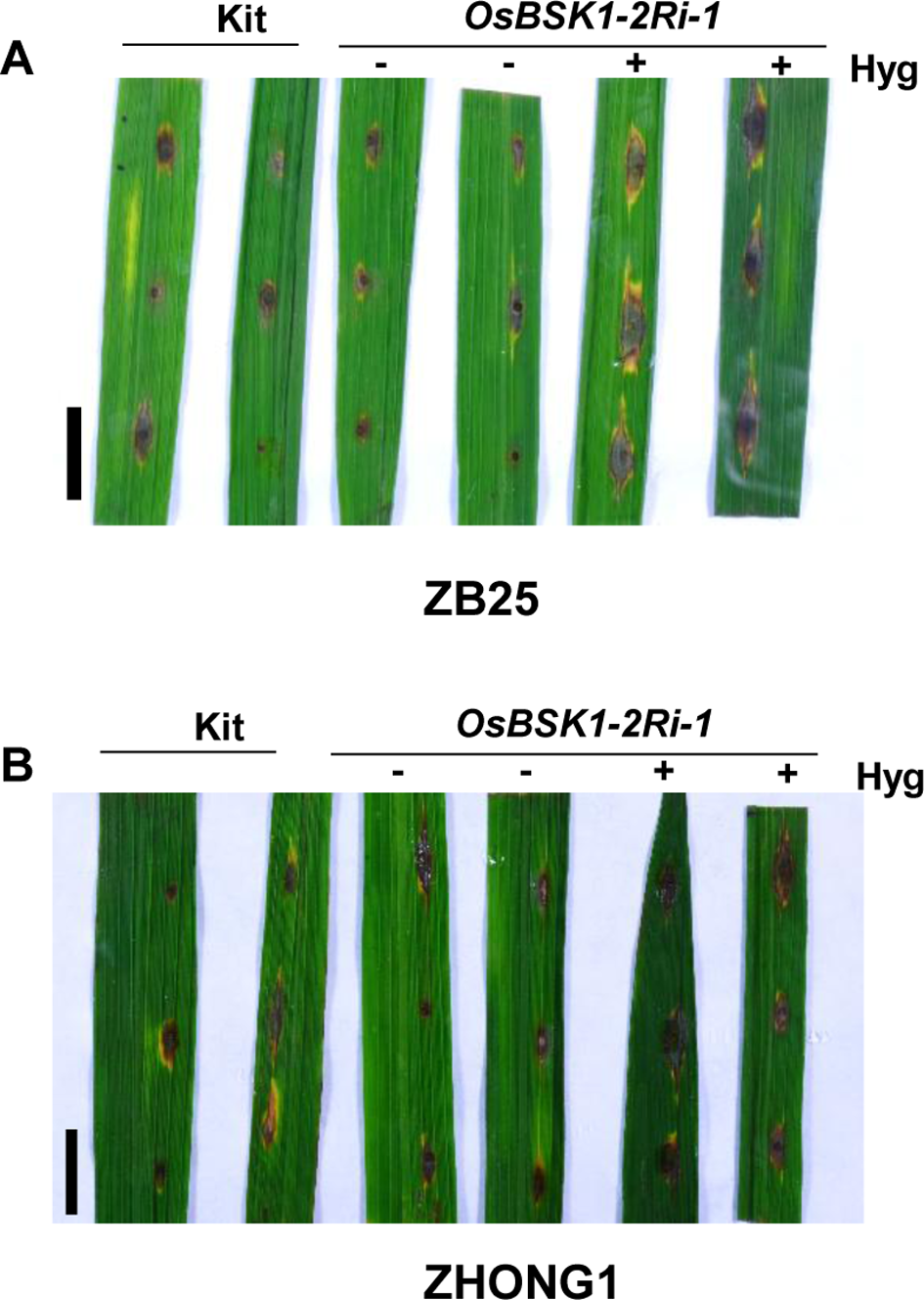

Supplement: FIGURE S3 — Co-segregation analysis on reduced resistance with the OsBSK1-2Ri transgene in plants. Segregants from the T1 progeny of transgenic OsBSK1-2Ri-1 line was analyzed for disease resistance post the inoculation with M. oryzae isolates, ZB25 (A) and ZHONG1 (B), respectively. [file Image_3.tif]

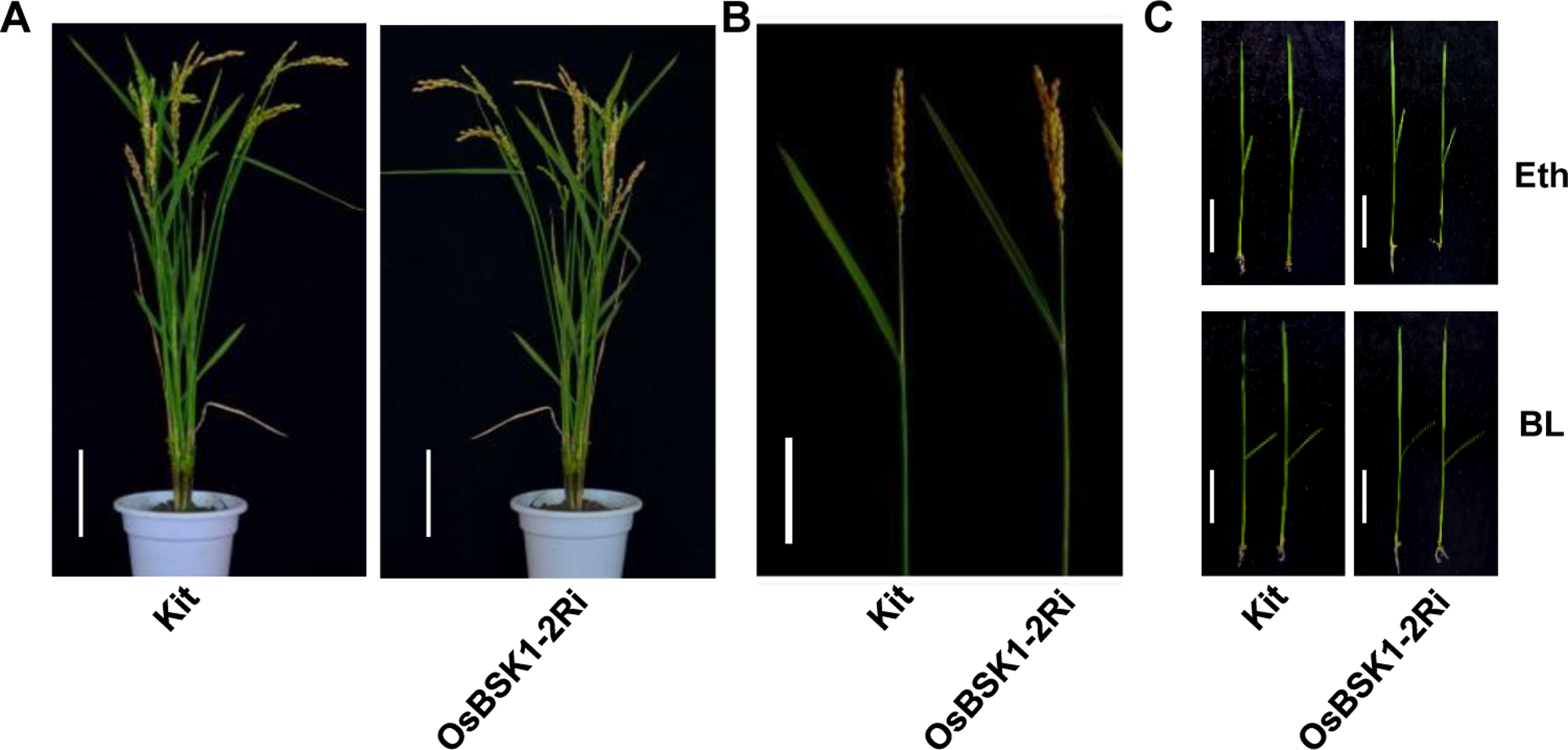

Supplement: FIGURE S4 — Silencing of OsBSK1-2 does not effect plant response to BRs obviously. (A) The gross morphological phenotypes of Kitaake and transgenic plants silenced for OsBSK1-2. Bars = 10 cm. (B) Photograph of a representative leaf of Kitaake (Kit) and OsBSK1-2Ri plants. Bars = 5 cm. (C) Photographs of representative seedlings showing the leaf angles for the Kitaakeand OsBSK1-2Ri plants after the mock-treatments (Upper panel) or BL treatment (Lower panel). Eth solution containing 2,4-epiBL (100 ng) was used for the BL treatment, whereas Eth alone was used for the mock treatment. [file Image_4.tif]
