# Supplementary material for: OsBSK1-2, an Orthologous of AtBSK1, Is Involved in Rice Immunity
Source: Front Plant Sci. 2017 Jun 21;8:908. doi: 10.3389/fpls.2017.00908 (PMC5478731; doi:10.3389/fpls.2017.00908)
Supplement: Supplementary file 5 [file Tables.DOCX]

**Supplemental Table 1: primers used to generate constructs**

| Names | Sequences (5’-3’) |
| --- | --- |
| OsBSK1Ri-F | CGCATGTAAAATAAATTGATG |
| OsBSK1Ri-R | GTTTGGAAAAGTATTCGC |

**Supplemental Table 2: primers used in q-PCR**

| Names | Sequences (5’-3’) |
| --- | --- |
| OsBSK1-qF | AGAAGCATCCAAGCAATA |
| OsBSK1-qR | TCCAAGCATCACATAAGAA |
| OsBSK2-qF | ATCTTGCTTCTCAGTGTT |
| OsBSK2-qR | GAGGTGCTTCTTCTTCAT |
| PR10b-qF | GTCGCGGTGTCGGTGGAGAG |
| PR10b-qR | ACGGCGTCGATGAATCCGGC |
| 04G10010-qF | AAATGATTTGGGACCAGTCG |
| 04G10010-qR | GATGGAATGTCCTCGCAAAC |
| OsUG-F | TTCTGGTCCTTCCACTTTCAG |
| OsUG-R | ACGATTGATTTAACCAGTCCATGA |
| Pot2-F | ACGACCCGTCTTTACTTATTTGG |
| Pot2-R | AAGTAGCGTTGGTTTTGTTGGAT |
